# WASH activities at two Ebola treatment units in Sierra Leone

**DOI:** 10.1371/journal.pone.0198235

**Published:** 2018-05-24

**Authors:** Michaela Mallow, Lee Gary, Timmy Jeng, Bob Bongomin, Miriam Tamar Aschkenasy, Peter Wallis, Hilarie H. Cranmer, Estifanos Debasu, Adam C. Levine

**Affiliations:** 1 International Medical Corps, Los Angeles, CA, United States of America; 2 The Payson Center for International Development at Tulane University, New Orleans, LA, United States of America; 3 Warren Alpert Medical School of Brown University, Providence, RI, United States of America; 4 Massachusetts General Hospital, Global Health, Global Disaster Response, Boston, MA, United States of America; University of Texas Medical Branch at Galveston, UNITED STATES

## Abstract

**Purpose:**

The 2014 outbreak of Ebola virus disease (EVD) in West Africa was the largest in history. Starting in September 2014, International Medical Corps (IMC) operated five Ebola treatment units (ETUs) in Sierra Leone and Liberia. This paper explores how future infectious disease outbreak facilities in resource-limited settings can be planned, organized, and managed by analyzing data collected on water, sanitation, and hygiene (WASH) and infection prevention control (IPC) protocols.

**Design/Methodology/Approach:**

We conducted a retrospective cohort study by analyzing WASH/IPC activity data routinely recorded on paper forms or white boards at ETUs during the outbreak and later merged into a database from two IMC-run ETUs in Sierra Leone between December 2014 and December 2015.

**Findings:**

The IMC WASH/IPC database contains data from over 369 days. Our results highlight parameters key to designing and maintaining an ETU. High concentration chlorine solution usage was highly correlated with both daily patient occupancy and high-risk zone staff entries; low concentration chlorine usage was less well explained by these measures. There is high demand for laundering and disinfecting of personal protective equipment (PPE) on a daily basis and approximately 1 (0–4) piece of PPE is damaged each day.

**Research limitations/Implications:**

Lack of standardization in the type and format of data collected at ETUs made constructing the WASH/IPC database difficult. However, the data presented here may help inform humanitarian response operations in future epidemics.

## Introduction

The outbreak of Ebola virus disease (EVD) outbreak in West Africa that began in 2014 is the largest and most devastating since the Ebola virus was first discovered in 1976.[1, 2] The World Health Organization (WHO) estimates there were over 28,000 suspected and confirmed cases and more than 11,000 deaths.[1–3] The epidemic affected countries around the world, but the hardest hit were three countries in West Africa: Guinea, Liberia and Sierra Leone.[2, 4, 5] The outbreak placed a significant strain on the region, which was already lacking a robust public health infrastructure, including appropriate Infection Prevention and Control (IPC) measures, critical water, sanitation and hygiene (WASH) supplies, accessible health care facilities and well trained health and infection control professionals.[3, 6, 7]

EVD is characterized by symptoms of fever, weakness and pain that may progress to internal and external bleeding, shock and death during the later stages of the infection.[8] Transmission of EVD can occur through broken skin or mucous membranes, when an individual has direct contact with the blood and/or bodily fluids of an Ebola positive patient.[9] As patients progress through the disease, they become increasingly infectious with higher viral loads and increased production of infectious bodily fluids.[10] Therefore, family members, caretakers and health care workers of Ebola patients are especially at risk for contracting and transferring the virus. In order to control this unprecedented outbreak, it was essential to stop transmission and end the spread of the disease in the most affected populations in West Africa. One of the most effective ways this was done was through providing care to sick patients in appropriate settings, such as Ebola treatment units (ETUs).

In order to run an ETU, strict IPC measures and an effective and robust WASH team focused on WASH activities proved essential to protect patients and staff. Core WASH activities in an ETU setting are based on clearly defined and promulgated protocols for all activities related to IPC. These activities include: sensitization of clinical and non-clinical staff working in and around the ETU to the highly infectious nature of EVD; establishing clear protocols; training all staff on these protocols; providing critical nonclinical support to staff and patients including sanitization of facilities; ensuring safe and proper disposal of contaminated material; ensuring proper and dignified burial of deceased patients; and overseeing the logistics and procurement of appropriate materials including personal protective equipment (PPE) and chlorinated water. PPE includes the various pieces of protective clothing worn to prevent staff in the ETU from coming in contact with bodily fluids which may contain the Ebola virus, including hoods, goggles, masks, coveralls, aprons, gloves, and boots. Chlorine is used for decontamination in the ETU setting, with 0.5% chlorine used for disinfecting equipment and surfaces and 0.05% for washing hands and disinfecting skin. The design of the ETU is also essential for proper IPC. ETUs are generally divided into a high-risk zone, which includes separate wards for patients with suspected and confirmed Ebola, and a low-risk zone, where clinical staff and ancillary activities such as food preparation and laundry are based. Staff would undergo a process of donning PPE in the low-risk zone before entering the high-risk zone, where they would complete their clinical or WASH activities and then undergo a process of doffing their PPE before returning to the low-risk zone.

Using data collected as part of operational procedures at two ETUs in Sierra Leone in 2014 and 2015, this study provides insight for the planning, organizing and managing of future infectious disease outbreak facilities in resource-limited settings. In particular, we utilize empirically collected data to provide estimates of chlorine, personal protective equipment (PPE), supply, and staffing needs in the context of managing an ETU in an EVD outbreak setting.

## Methods

### Study design and setting

This retrospective cohort study includes data on key WASH/IPC activities carried out in two ETUs in Sierra Leone operated by International Medical Corps (IMC) between December 2014 and December 2015 as part of its comprehensive response to the EVD epidemic in West Africa. Data was collected from the Makeni ETU, located in the Bombali District of Sierra Leone and the Kambia ETU, located in the Kambia District of Sierra Leone. The Makeni ETU had a maximum daily patient occupancy of 58 patients and maximum monthly staff levels of 300 personnel at the height of the epidemic; the smaller Kambia ETU had a maximum daily patient occupancy of 16 patients and maximum monthly staff levels of 150 personnel. Both utilized standard ETU design, as described above, with high and low-risk zones, though the Makeni ETU was built *de novo* while the Kambia ETU utilized a previously existing health facility. As no individual patient data was collected for this study, we did not seek formal ethical approval. No additional approvals or permits were required under Sierra Leone law for this retrospective research. All data for this study has been made freely available to the public.

### Data collection

Data on all WASH/IPC activities were recorded by a WASH Officer at each ETU as part of routine WASH/IPC activities on a WASH/IPC logbook in two ways: (1) inventory and status of supplies and activities by shift and (2) “briefing-debriefing” sessions at the start of each shift.

1. Inventory and status of supplies and activities by shift included:
  - Water: quantity used in the low and high risk zones; concentration (fresh, 0.5% chlorine solution, 0.05% chlorine solution) used in the low and high risk zones; number of pumping hours; number and location of water taps and tanks; and details on any repairs and/or maintenance required for the water distribution network
  - Chlorine: stock reports including types of chlorine and protective equipment (nitrile inner gloves, heavy duty rubber gloves, chemical mask, scrubs/gown, heavy waterproof apron and boots) required to handle chlorine; quantity used to dose tanks with 0.5% (chlorine water tanks) or 0.05% (fresh water tanks) chlorine concentrations; and mixing and refilling time and contact time (at least 30 minutes for chlorine to be able to effectively kill/inactivate pathogenic organisms) of the chlorine treated water
  - PPE consumption: stock reports including quantity requested from warehouse to donning/dressing room; size and specification for each type of PPE used in the low and high risk zones including whether the type of PPE was disposable or re-usable; record of damages; record of disposable PPE taken for incineration; record of re-usable PPE to be disinfected, cleaned and dried; and record of disinfected, cleaned and dried PPE brought back to the donning/dressing room
  - Laundry: quantity brought in from and returned to donning/dressing room; and quantity of detergent and soaps used for washing
  - Waste management: quantity (in kilograms) of waste produced in the low and high risk zones; number of waste bags used; number of sharps collected into sharps boxes and properly disposed of into sharps pit; number of buckets for wet symptoms used and contents properly disposed of into latrines; and liquid waste from laundry and wards channeled into soak pits within the high risk zone
  - High risk zone activities: number of wards disinfected; number of patient beds disinfected; number of resting areas disinfected; number of bathrooms (e.g. toilets, showers, etc.) disinfected; and number of repairs to plumbing connections
  - Low risk environmental cleaning: office cleaning; picking up trash; cleaning of toilets and bathrooms; and cleaning of drainage
  - Staffing: number of staff assigned to each 8-hour shift; number of staff supervising (should be one officer and one shift supervisor); and number of staff in charge of waste, laundry, chlorinators, sprayers, dead body management, and high risk zone hygienists
2. At the start of each shift, all staff assembled into the hygienist room for a brief presentation by the outgoing shift. In this presentation, areas of focus were highlighted, tracking forms handed over to incoming shift staff and the rigorous procedures in place for staff and patient safety while working at the ETU reinforced.

Initially, data were collected on paper forms or white boards depending on ETU procedures. Data were later entered into separate electronic databases at each ETU by WASH Officers and Managers on a weekly basis for all data collected from the low risk zone and on a daily basis for all data collected from the high risk zone. Later, these data were combined into a unified database by IMC staff.

### Variables of interest

The primary variables of interest for WASH/IPC activities in ETUs were 0.05% (low concentration) and 0.5% (high concentration) chlorine solution consumption, waste bags incinerated, beds/cubicles disinfected, low risk zone and high risk zone staff entries, PPE used/damaged (scrubs, goggles, boots, aprons, coveralls, hoods, masks, gloves) and daily patient occupancy.

### Data analysis

Descriptive statistics were calculated for the primary variables of interest. We conducted univariable and multivariable linear regression analyses to examine differences in amounts of chlorine used, WASH activities performed and PPE used by both daily patient occupancy and daily high-risk zone (HRZ) staff entries, in order to develop a predictive model for future usage of chlorine based on patient and staffing considerations in an ETU. The adjusted R squared statistic was used to estimate the variability in data explained by each model. In all cases, a p value less than 0.05 was considered significant. Data analyses were conducted in STATA 13 (StataCorp, TX, USA).

## Findings

The full International Medical Corps WASH/IPC activities database consisted of information collected from two ETUs in Sierra Leone over the course of 369 days. Approximately one-third of the data were from the Makeni ETU in Bombali District, covering the period of December 2014 to April 2015, while the rest of the data were from the Kambia ETU in Kambia District, covering the period of April 2015 to December 2015.

Table 1 shows median daily values for key operational variables, including daily patient occupancy, chlorine solution consumption, high-risk zone staff entries, and waste management activities, combined for the Makeni and Kambia facilities.

**Table 1:** Key variables in an ETU setting, Kambia and Makeni, Sierra Leone, December 2014 to April 2015.

	Key Variables*	Median (IQR)
Daily patient occupancy		6 (3–11)
Chlorine Solution Consumption**		
	Low Chlorine (0.05% cl)	2000 (1600–2340)
	High Chlorine (0.5% cl)	2490 (1520–4200)
High Risk Zone Entries		
	Medical Staff	14 (7–20)
	WASH Staff	16 (8–29)
	Total Staff	31 (15.5–47.5)
WASH Management Activities		
	Incinerated Bags (HRZ)	14 (7–22)
	Incinerated Bags (LRZ)	20 (17–24)
	Disinfected Cubical Beds	4 (2–12)

*Per day

**Liters

### High concentration (0.5%) chlorine solution usage

**Fig 1:**
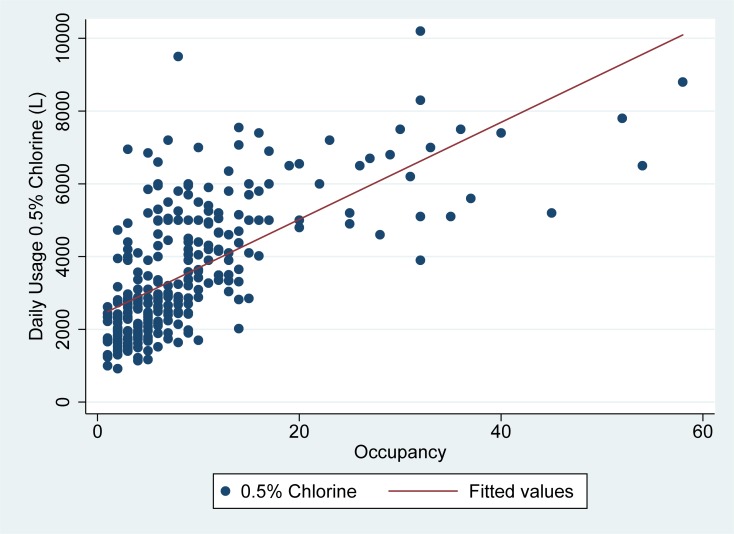
Usage of high concentration (0.5%) chlorine vs. occupancy in Sierra Leone ETUs. Fig 1 demonstrates daily patient occupancy (x-axis) as compared to the usage of high concentration (0.5%) chlorine in liters (y-axis) for the two ETUs studied.

**Fig 2:**
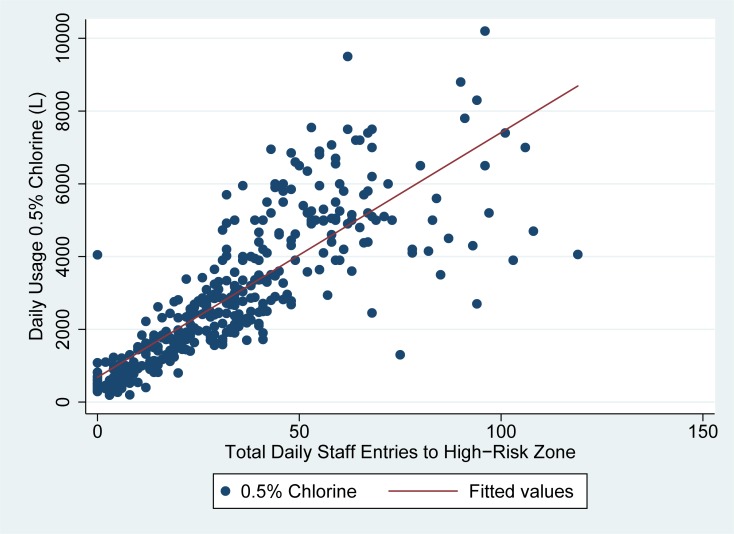
Usage of high concentration (0.5%) chlorine vs. total hrz staff entries in Sierra Leone ETUs. Fig 2 demonstrates the number of times staff entered the high-risk zone each day as compared to the total usage of high concentration (0.5%) chlorine in liters (y-axis) for the two ETUs studied.

As seen in Figs 1 and 2, the usage of high concentration chlorine (0.5%) solution was highly correlated with both daily patient occupancy and high-risk zone staff entries in linear regression analyses.

High risk zone staff entries explained about 66% of the variability in daily 0.5% chlorine solution usage, while daily patient occupancy explained about 44% of the variability in daily 0.5% chlorine usage. In linear regression analysis, 67 liters (95% CI: 62–72) of 0.5% chlorine were used on average for each high risk zone staff entry on a given day, while 133 liters (95% CI: 116–151) of 0.5% chlorine was used per admitted patient per day in the ETU. (Table 2)

**Table 2:** Multivariate linear regression analysis, Kambia and Makeni, Sierra Leone, December 2014 to April 2015.

				Occupancy			High Risk Staff Zone Entries			
				95% CI				95% CI		
			Coefficient	Lower	Upper	p	Coefficient	Lower	Upper	p
Chlorine										
	0.5% Chlorine		133.45	116.06	150.84	<0.01	67.30	62.34	72.27	<0.01
	0.05% Chlorine		22.19	12.75	31.63	<0.01	18.67	16.02	21.32	<0.01
Incinerated										
	Bags Incinerated		0.42	0.24	0.60	<0.01	-	-	-	-
PPE										
	Disinfected									
		Cubical Beds Disinfected	0.91	0.82	1.00	<0.01	-	-	-	-
		Goggles Disinfected	-	-	-	-	1.26	1.15	1.37	<0.01
	Laundered									
		Heavy Duty Gloves Laundered	-	-	-	-	0.95	0.83	1.08	<0.01
		Scrubs Laundered	-	-	-	-	1.92	1.63	2.21	<0.01
		Aprons Laundered	-	-	-	-	1.07	0.93	1.21	<0.01
		Boots Laundered	-	-	-	-	1.58	1.14	2.01	<0.01
	Used									
		Coveralls Used	-	-	-	-	0.86	0.79	0.93	<0.01
		PPE Masks Used*	-	-	-	-	1.03	0.98	1.08	<0.01
		Hoods Used	-	-	-	-	0.99	0.98	1.00	<0.01

*N95 Masks

### Low concentration (0.05%) chlorine solution usage

**Fig 3:**
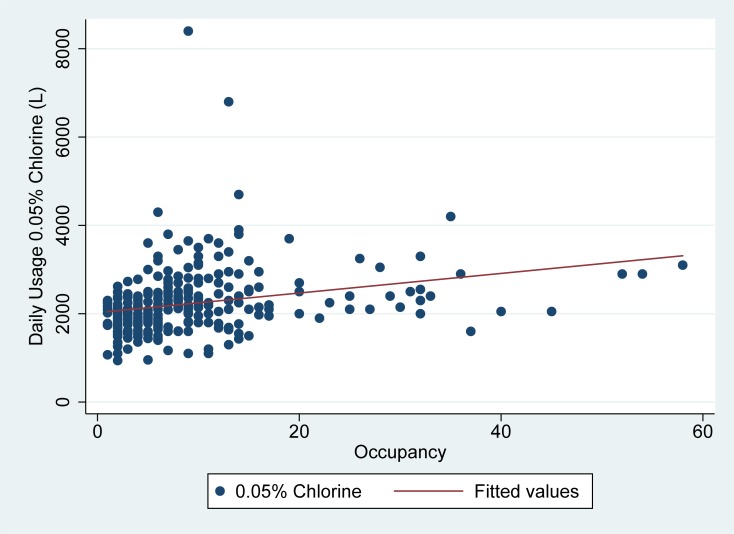
Usage of low concentration (0.05%) chlorine vs. occupancy in Sierra Leone ETUs. Fig 3 demonstrates daily patient occupancy (x-axis) as compared to the usage of low concentration (0.05%) chlorine in liters (y-axis) for the two ETUs studied.

**Fig 4:**
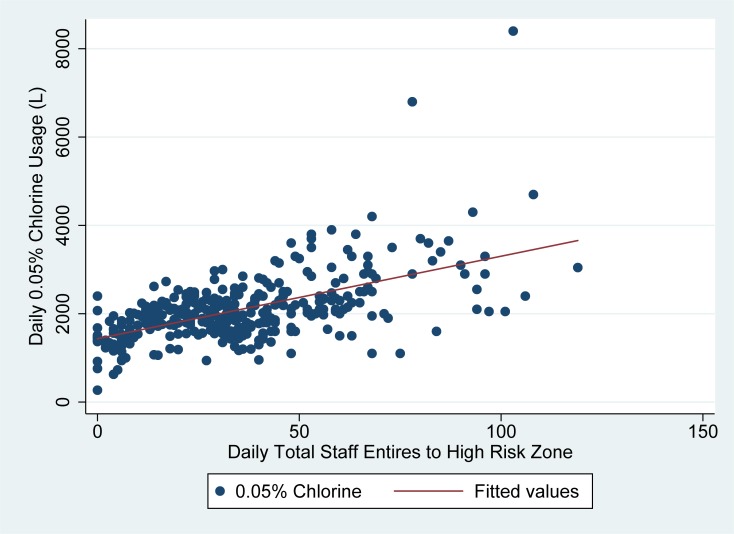
Usage of low concentration (0.05%) chlorine vs. total HRZ staff entries in Sierra Leone ETUs. Fig 4 demonstrates the number of times staff entered the high-risk zone each day as compared to the total usage of low concentration (0.05%) chlorine in liters (y-axis) for the two ETUs studied.

As seen in Figs 3 and 4, the usage of low concentration (0.05% chlorine) was less well explained by daily patient occupancy and high risk zone staff entries. In linear regression models, high risk zone staff entries explained about 35% of the variability in daily 0.05% chlorine usage, while daily patient occupancy explained just 7% of the variability in daily 0.05% chlorine usage. In linear regression analysis, 19 liters (95% CI: 16–21) of 0.05% chlorine was used on average for each high risk zone staff entry on a given day, while 22 liters (95% CI: 13–32) of 0.05% chlorine was used per admitted patient per day in the ETU. (Table 2)

### Differences in chlorine usage by ETU

Even controlling for daily patient occupancy and high risk zone staff entries, there were significant differences in chlorine usage by ETU. In multivariable linear regression, the Makeni ETU used 1982 (95% CI: 1659–2305) additional liters of 0.5% chlorine per day as compared to the Kambia ETU. There were no significant differences in the use of 0.05% chlorine per day by ETU after controlling for daily patient occupancy and high risk zone staff entries.

### WASH activities

Table 2 also demonstrates the relationship between daily patient occupancy and key WASH activities, including the number of cubicles cleaned and waste bags incinerated on a given day. As expected, just under 1 cubicle per day was cleaned per patient in the ETU, while about 0.4 bags of hazardous and medical waste were incinerated each day per patient in the ETU.

### PPE

Table 3 demonstrates the high demand for laundering and disinfecting of PPE on a daily basis in an ETU setting. Approximately 126 (108–162) scrubs were laundered each day, along with 200 (170–233) boots and 59 (43–72) aprons. Our data suggests that approximately 1 (0–4) piece of PPE is damaged each day and 2 (2–3) sprayers must be repaired each day.

**Table 3:** Daily PPE usage in an ETU setting, Kambia and Makeni, Sierra Leone, December 2014 to April 2015.

Key Variables		Median (IQR)
Disinfected		
	Goggles	37 (19–50)
	Latrines	3 (3–4)
	Wards	3 (2–3)
Laundered		
	Scrubs	126 (108–162)
	Aprons	59 (43–72)
	Heavy Duty Gloves	52 (39–64)
	Boots	200 (170–233)
Used		
	Coveralls	19 (7.5–30.5)
	N95 Masks	20 (8–31)
	Hoods	20 (9–31)
Damaged/Repaired		
	Total PPE Damaged	1 (0–4)
	Sprayers Repaired	2 (2–3)

## Discussion

Like other infectious disease interventions, EVD outbreak interventions require efficiently designed and operated treatment facilities in order to ensure a low risk of nosocomial infection and easy to maintain monitoring WASH/IPC practices. [11] Our study highlights parameters that are key in designing and managing a treatment facility for future infectious disease outbreaks in resource-limited settings.

Our data shows that high concentration (0.5%) chlorine usage was linked to both staff high risk zone entries and patient population, but correlated much better with high risk zone staff entries. Decisions on how much high concentration chlorine should be ordered will therefore be based on how many staff will be rounding over how many rounds daily as opposed to the more unpredictable measure of a patient population. Low concentration chlorine usage was less well explained by daily patient occupancy and high risk zone entries as all staff and patients throughout the ETU–in the low-and-high risk zones–were utilizing low concentration chlorine solution for activities such as bathing, handwashing, laundry, and kitchen use (e.g. utensils). We also noted significant variability in chlorine usage between the two ETUs studied. This difference may be explained by the larger catchment area and ambulance fleet utilized by the Makeni facility, which would have required significant high concentration chlorine usage to disinfect ambulances after each trip. This is an important logistical point, which should also be taken into account during ETU operational planning.

Our data demonstrated that WASH activities such as the number of cubicles cleaned and the number of waste bags incinerated daily is correlated to daily patient occupancy. This information is helpful for WASH staff planning purposes with regards to incineration needs within ETUs and allows for an incineration schedule based on waste volume and replenishment of essential waste bags both in warehouse and in the high risk zone.

Laundering and disinfecting PPE on a daily basis is extremely important to estimating the number of items needed for laundering. The number of PPE damaged gives a sense of how often each type of PPE is damaged and how often we can expect to replace items on a daily basis. Damaged sprayers cannot be overlooked as they are vital to IPC/WASH protocols and on average, 2–3 are damaged daily. Timely replacement is vital, which can be difficult due to overwhelmed local markets, which might not normally supply this high quality product.

### Limitations

One of the greatest challenges in building our database was the lack of standardization in the data collected across different ETUs. Despite being managed by the same organization, the various ETUs collected different types of WASH/IPC data in variable formats and in some cases the types of data collected changed over time. This was due to a variety of factors, including the emergent nature of the epidemic, the lack of time to agree upon and disseminate standardized data collection forms and the lack of prior empiric evidence on which data elements were most important to collect in the context of operating an ETU.

The severe logistical constraints related to collecting data in a treatment facility tailored to a highly contagious and virulent disease such as Ebola cannot be overemphasized. The majority of IPC/WASH data were collected in the ETU’s high risk zone. Therefore, staff collecting the data were either dressed in full PPE, which limited both their movements and the time they could spend collecting and recording information, or they recorded information by recall on a whiteboard after exit from the high risk zone, doffing and rest/rehydration. This may have led to recall bias. In the future, other solutions such as electronic databases access through hand held tablets could be considered for more efficient means of collecting data. [12]

## Conclusion

Even for organizations and individuals with significant humanitarian logistics and supply chain experience, the unique factors involved in managing an ETU during an EVD outbreak require special consideration. The key findings from this study, as well as lessons learned with regards to data collection, will inform the planning, organizing, and managing of ETUs in future Ebola or other infectious disease outbreak. In particular, this research provides estimates on the amount of chlorine and personal protective equipment required to manage an ETU during a future Ebola epidemic, based on the anticipated size and staffing of the ETU. The manuscript also provides recommendations for improving operational data collection in future similar humanitarian emergencies, in order to contribute to continuous learning and improvement.

## Supporting information

S1 Fig The original dataset used for analyses. (XLSX)
